# Breakdown of intention-based outcome evaluation after transient right temporoparietal junction deactivation

**DOI:** 10.1038/s41598-023-28293-w

**Published:** 2023-01-23

**Authors:** Junfeng Zhang, Sai Sun, Chengyan Zhou, Yaochun Cai, Hao Liu, Zhaoyang Yang, Rongjun Yu

**Affiliations:** 1 Research Base of Traditional Chinese Medicine Syndrome, Fujian University of Traditional Chinese Medicine, Fuzhou, China; 2 Frontier Research Institute for Interdisciplinary Sciences, Tohoku University, Sendai, Japan; 3 Research Institute of Electrical Communication, Tohoku University, Sendai, Japan; 4 School of Psychology, South China Normal University, Guangzhou, China; 5 Department of Psychology, Guangzhou University, Guangzhou, China; 6 Department of Management, Marketing, and Information Systems, School of Business, Hong Kong Baptist University, Kowloon Tong, HKSAR, Hong Kong, China

**Keywords:** Social behaviour, Decision

## Abstract

People judge the nature of human behaviors based on underlying intentions and possible outcomes. Recent studies have demonstrated a causal role of the right temporoparietal junction (rTPJ) in modulating both intention and intention-based outcome evaluations during social judgments. However, these studies mainly used hypothetical scenarios with socially undesirable contexts (bad/neutral intentions and bad/neutral outcomes), leaving the role of rTPJ in judging good intentions and good outcomes unclear. In the current study, participants were instructed to make goodness judgments as a third party toward the monetary allocations from one proposer to another responder. Critically, in some cases, the initial allocation by the proposer could be reversed by the computer, yielding combinations of good/bad intentions (of the proposer) with good/bad outcomes (for the responder). Anodal (n = 20), cathodal (n = 21), and sham (n = 21) transcranial direct current stimulation (tDCS) over the rTPJ were randomly assigned to 62 subjects to further examine the effects of stimulation over the rTPJ in modulating intention-based outcome evaluation. Compared to the anodal and sham stimulations, cathodal tDCS over the rTPJ reduced the goodness ratings of good/bad outcomes when the intentions were good, whereas it showed no significant effect on outcome ratings under unknown and bad intentions. Our results provide the first evidence that deactivating the rTPJ modulates outcome evaluation in an intention-dependent fashion, mainly by reducing the goodness rating towards both good/bad outcomes when the intentions are good. Our findings argue for a causal role of the rTPJ in modulating intention-based social judgments and point to nuanced effects of rTPJ modulation.

## Introduction

People judge the nature of individual behaviors not only relying on the outcomes caused by their behaviors but also on the underlying intentions. Studies have shown a transition from outcomes-oriented to intention-based moral judgment from children to adults in the normal population^1–3^. Using functional magnetic resonance imaging (fMRI), accumulating studies have consistently shown the involvement of the right temporoparietal junction (rTPJ) in intention and outcome processing during social judgments^4–7^. The functional selectivity of the rTPJ also plays a crucial role in representing the increasing intention attribution in children from 6 to 11 years old^2^. Structural and functional abnormality in the rTPJ may characterize the deficits in integrating intention into outcome evaluation among autism spectrum disorders (ASD)^8,9^. However, these findings do not support causal claims about the rTPJ function.

Transcranial direct current stimulation (tDCS) or transcranial magnetic stimulation (TMS), as non-invasive brain stimulation technique, allows it possible to enhance or disrupt cortical excitability artificially and transiently and further examine the causal role of the rTPJ in intention/outcome attribution during social judgment^10^. The Anodal stimulation using tDCS is often intended to enhance while cathodal stimulation is intended to decrease the cortical excitability^11–13^; see also the discussion about polarity-depended excitatory effects of tDCS^14^. Similarly, the high-frequency TMS (e.g., 10 Hz) is often designed to enhance cortical excitability, while the low-frequency TMS (e.g., 1 Hz) is often used to inhibit cortical activity^15^. Both cortical excitability and inhibition induced by stimulation have long-lasting effects^13,16,17^.

Using a low-frequency (1 Hz) TMS, Young et al., demonstrated that inhibiting the activity of rTPJ enhanced the moral permissibility of attempted (*negative intention*) but failed harms (*neutral outcomes*)^15^, suggesting a decrease of intention weighting after disrupting the rTPJ. Using a similar paradigm, Sellaro et al. found that participants under a 1.0 mA anodal tDCS over the rTPJ assigned higher permissible ratings to accidental harms (*neutral intention, negative outcome*)^18^, suggesting that activating the rTPJ increases the weighting of intention. These two pioneering studies have consistently indicated the causal role of the rTPJ in modulating intention-based social judgments with activating the rTPJ tends to enhance the weighting of intention, while deactivating the rTPJ shows the opposite.

Besides the polarity of the tDCS stimulation, another study examined the effects of stimulation intensity on moral judgments, in which 1.5 mA cathodal tDCS over the rTPJ decreased moral appropriateness of accidental harm (*neutral intention, negative outcome*) whereas 0.8 mA increased the appropriateness ratings^19^. These results suggested that the modulatory effects of the tDCS on intention attribution are intensity-dependent, with stronger (negative) intensity tends to decrease the intention weighting, whereas weaker (negative) intensity increase the intention weighting. Interestingly, administrating 2.0 mA cathodal tDCS over the rTPJ with simultaneous anodal over the left TPJ increased permission rating of neutral acts with attempted harm (*negative intention, neutral outcome*)^20^. Permitting attempted harm suggests that the negative intention is not being taken full consideration when inhibiting the rTPJ or exciting the left TPJ. In contrast, anodal over the rTPJ while cathodal tDCS over the left TPJ decreased permission rating of intentional harm (*negative intention, negative outcome*)^20^. This finding suggests that exciting rTPJ or inhibiting the left TPJ promotes the consideration of intention when the outcome is negative. Again, this line of research has consistently demonstrated a key role of the rTPJ in modulating intention-based attribution during social judgment, with anodal stimulation over the rTPJ tends to decrease the consideration of bad intentions, and cathodal stimulation over the rTPJ tends to increase the permission of harmful behaviors with bad intentions.

Notably, the aforementioned studies only considered socially undesirable scenarios—bad/neutral intentions and bad/neutral outcomes^4,18–20^, leaving the question of whether the rTPJ plays a similar role in judging socially desirable events underexplored. Though one study has shown that cathodal tDCS over the rTPJ reduced judgment time for helpful intention-based scenarios compared to sham stimulation, no changes were shown on the rating scores^21^. Reward-credit assignment is critical to maintain cooperation and motivate prosocial activities. Appropriate employee recognition encourages organizational citizenship behavior and giving well-deserved praise to workers is essential to an outstanding workplace. To further demonstrate the role of the rTPJ in intention-based attribution under socially desirable situations, we introduced a modified dictator game in which participants were instructed to make judgments as a third party based on the monetary allocations from one proposer to another responder. Previous studies have demonstrated comparable and asymmetry processing of positive and negative moral behaviors during a third-party judgement task^5,22–24^. Critically, in some cases, the initial allocation by the proposer could be reversed by the computer, yielding combinations of good/bad intention (of the proposer) with good/bad outcome (for the responder)^25^. A good intention/outcome means giving the other party a relatively large proportion of the pie, usually more than 50%, while a bad intention/outcome means proposing a self-advantage offer that gives the other party small share, e.g., below 50%. In each trial, participants were asked to act as an interest-free third party and make goodness judgment of each proposer’s allocation that involves intention and real outcome. Behaviorally, we are interested in how normal adults make intention-based social judgements under mixed scenarios with both good and bad intentions/outcomes, and further whether (and how) transient tDCS stimulation over the rTPJ may modulate intention-based outcome evaluation.

To approach the above two questions, a combined behavioral and tDCS study was performed among 62 participants, who randomly underwent anodal (n = 20), cathodal (n = 21), and sham (n = 21) tDCS over the rTPJ. Based on our previous work, we hypothesized that a similar pattern of intention-oriented attribution could be observed in a mixed scenario with good and bad intentions/outcomes in a baseline condition. Moreover, the anodal tDCS and cathodal tDCS may show differentially modulatory effects with anodal tDCS may increase intention attribution while the cathodal may decrease it.

## Materials and methods

### Participants

In a single-blinded, sham-controlled design, sixty-two college students (20.42 ± 1.9 years, 49 females) were recruited and randomly assigned to one of the three stimulation groups with anodal (n = 21), cathodal (n = 20), or sham (n = 21). We set the sample size based on previous studies on the effects of rTPJ stimulation on the intention processing ranging from 4 to 20 participants in each stimulation group^15,18–20^. Groups did not differ in gender (anodal (male/female): 5/16, cathodal: 3/17, and sham: 5/16; Pearson’s *χ*^2^ = 0.635, *p* = 0.728). Participants reported no history of neurological or psychiatric disorders. The study was approved by the Institutional Review Board (IRB) of the department of psychology at South China Normal University. All experiments were performed in accordance with relevant guidelines and regulations. All participants gave their informed consent prior to the experiment and were financially compensated for their participation.

### Experimental paradigm and procedure

**Figure 1:**
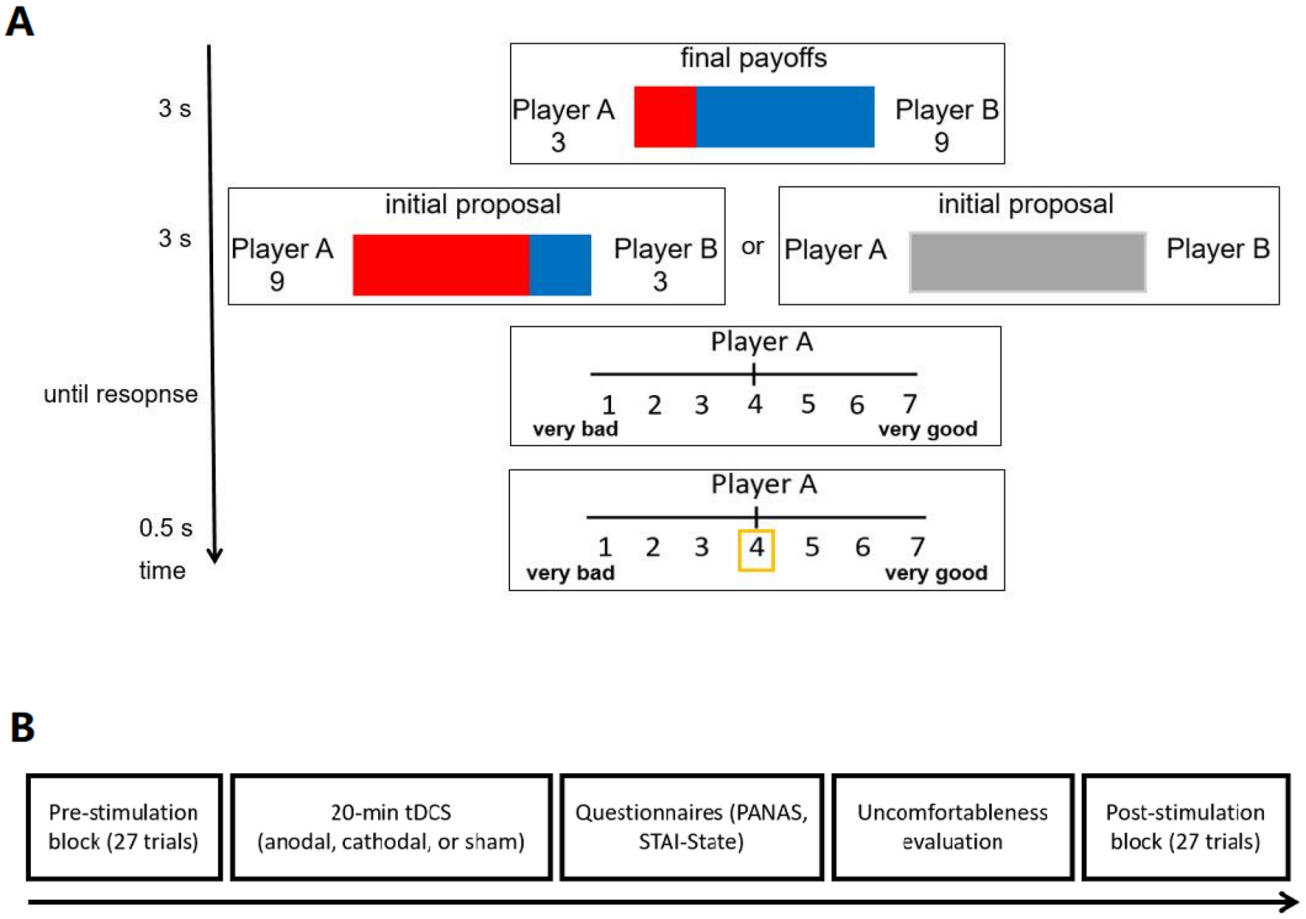
(**A**) An illustration of one trial of the social judgment task. The final payoffs for both Player A (the proposer) and Player B (the responder) were presented at the beginning of each trial. After that, in the certain trails, Player A’s initial proposal was revealed. In unknown intention trails, the initial proposal was not revealed (all grey). The example here showed a final payoff of 3¥ (about $0.5) for the proposer, and 9¥ (about $1.5) for the responder (good outcome). The final payoffs were a result of computer reverse procedure as the initial offer was 9¥ (about $1.5) for the proposer and 3¥ (about $0.5) for the responder (bad intention). A seven-point goodness rating scale (1 = very bad, 7 = very good) was employed to indicate the judgment of Player A’s behavior. (**B**) Illustration of the whole procedure.

A modified dictator game (see also Ref.^25^) was introduced in which participants were instructed to make goodness judgments as a third party toward the monetary allocations from one proposer to another responder (see Fig. 1A). Each trial has a total allocation of 12 ¥ (about $2). The offer from the proposer has 9 levels range from 2 to 10 ¥ with an increment of 1 ¥. Correspondingly, the responder will get an offer range from 10 to 2 ¥. The distribution of initial offer indicates the intention level, with a good intention means proposing an offer that is beyond the averaged distribution for the responder (i.e., 2/3/4 for the proposer-8/7/6 for the responder), while a bad intention means proposing an unfair offer that is far below the averaged distribution for the responder (i.e., 8/7/6 for the proposer-2/3/4 for the responder). A good outcome means the real offer is beyond the averaged distribution (i.e., 6/7/8 ¥), while a bad outcome means the offer that is far below the averaged distribution (i.e., 2/3/4 ¥). Importantly, the proposers’ offers could be reversed or not by a computer with a 50% chance The final payoffs were yielded after the reversal procedure. There were four combinations from two types of intentions of the proposer and two types of outcomes for the responder. Specifically, in the reversal condition, the offers/payoffs for the proposer and the recipient were exchanged; while in the non-reversal condition, the initial offers were the same as final payoffs. We also included unknown intention conditions in which the initial proposals were not shown to participants. In that case, there would be 3 intention conditions (good/unknown/bad) together with 2 outcomes (good/bad). Participants were asked to make goodness judgments as a third party toward the combinations of initial offers and final payoffs using a seven-point rating scale (1 = very bad, 7 = very good). Unbeknown to participants, all the allocations were experimentally manipulated, although participants were led to believe that proposers and responders were real. The instructions were delivered verbally in Mandarin.

All participants underwent two blocks, a pre-stimulation (baseline) block, and a post-stimulation block, with 27 trials (3 intention conditions × 9 offer levels) in each block. The pre-stimulation block was presented without any tDCS stimulation. The post-stimulation block followed immediately after a 20-min tDCS stimulation (anodal, cathodal, or sham). Subjective ratings on anxiety^26^, positive and negative affect^27^, and uncomfortable feelings after tDCS stimulation were collected to check the potential influence or subjective bias toward different stimulation (Fig. 1B).

### tDCS protocol

**Figure 2:**
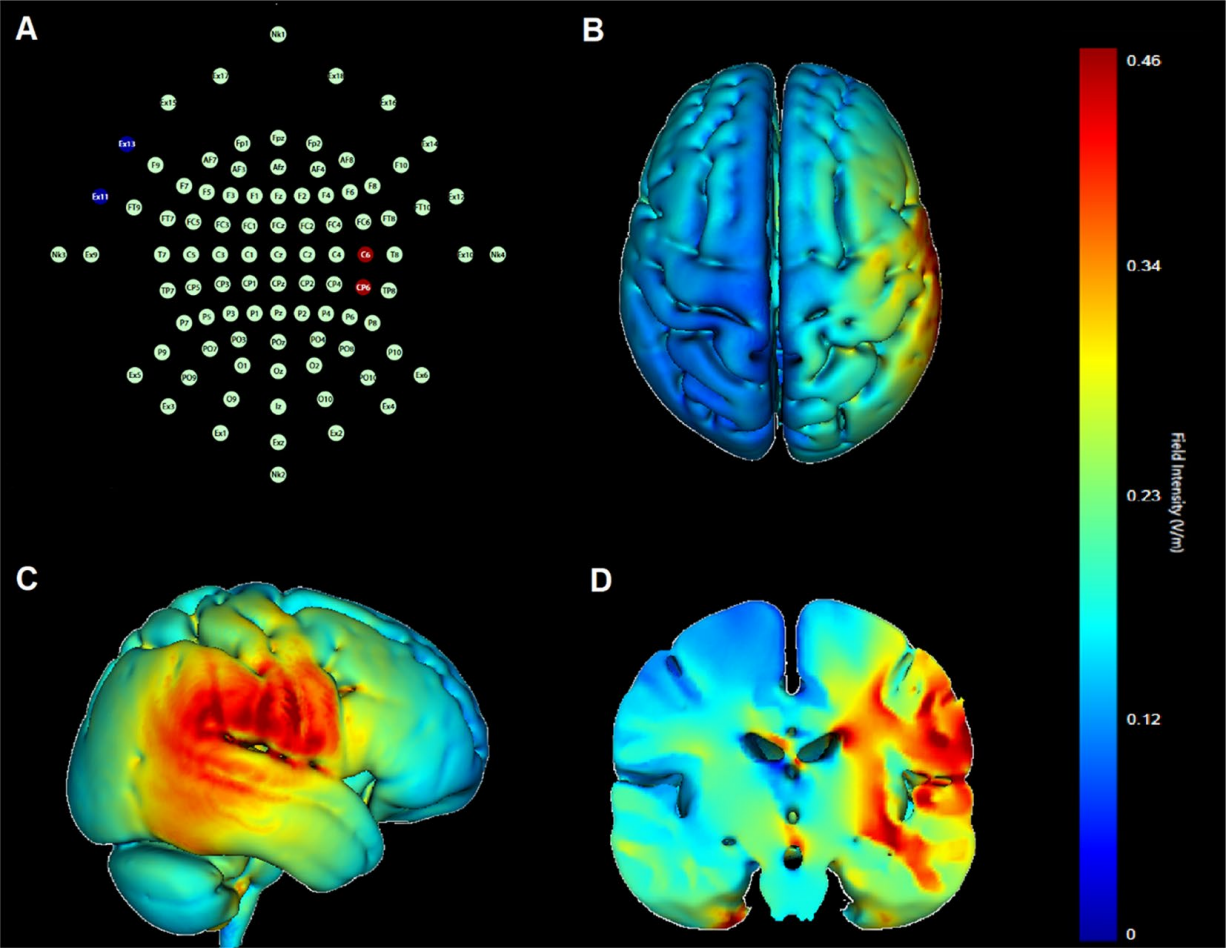
(**A**) An illustration of the stimulating electrodes positions based on the international 10–20 EEG system. (**B**–**D**) Simulation of the normalized electric field distribution in the brain: (**B**) superior view, (**C**) lateral view, (**D**) coronal view.

The stimulation was administered through a battery-driven constant current stimulator developed by Soterix Medical, America. The stimulation was induced through a saline-soaked pair of surface sponge electrodes. Both anodal and cathodal electrodes were 5 cm × 7 cm in size. To stimulate the rTPJ, the anodal or cathodal electrode was placed between CP6 and C6 according to the international 10–20 EEG system^28^, while the reference electrode was positioned over the left cheek^29^ (Fig. 2A). Simulation of the electric field distributions (shown in Fig. 2B–D) was performed based on a standard MNI 152 template (Montreal Neurological Institute, International Consortium for Brain Mapping) using HD-Explore™ 6.0 (https://soterixmedical.com/research/software/hd-explore). Although the optimal stimulation parameters remain to be elucidated, some studies have suggested tDCS with the intensity of 2.0 mA and the duration of 15–30 min can yield relevantly long-lasting changes in cortical excitability^16^, cerebral blood flow^17^, as well as changes in moral cognition and behavior^30^. Here, a constant current of 2.0 mA (current density of 0.057 mA/cm^2^) was delivered for 20 min. All stimulation conditions (anodal, cathodal, or sham) have both fade in and fade out period of 30 s. For sham stimulation, the electrodes were placed in the same positions as for the anodal/cathodal stimulation, but the stimulation only lasted for the initial 30 s. This method of sham stimulation has been proven to be reliable^31,32^. Moreover, previous studies have confirmed that participants were blind to stimulation conditions^31,33,34^. In our study, the electrode montage and adopted tDCS parameters were identical to those that have successfully modulated cortical excitability of the rTPJ^28,35,36^.

### Data analysis

All analyses were performed using SPSS Version 23. The allocation amounts were categorized into three conditions: good (more than 6 ¥), fair (6 ¥) and bad (less than 6 ¥), which was also indicated in another paper^25^. We firstly checked the intention-based social judgements towards various intention and outcome combinations in the baseline condition. Then, to evaluate the effects of tDCS on intention-based outcome evaluation, we then compared the rating difference between pre-tDCS and post-tDCS across three different stimulation methods. A four-way repeated measure ANOVA on social judgments was performed with intention (good vs. unknown vs. bad), outcome (good vs. bad), and phase (pre-tDCS vs. post-tDCS) as within-subject factors, stimulation group (anodal, cathodal, sham) as a between-subjects factor. A Greenhouse–Geisser correction for sphericity was used if applicable. The significance was reported if the *p* values survive below or equal to *0.05*.

## Results

**Figure 3:**
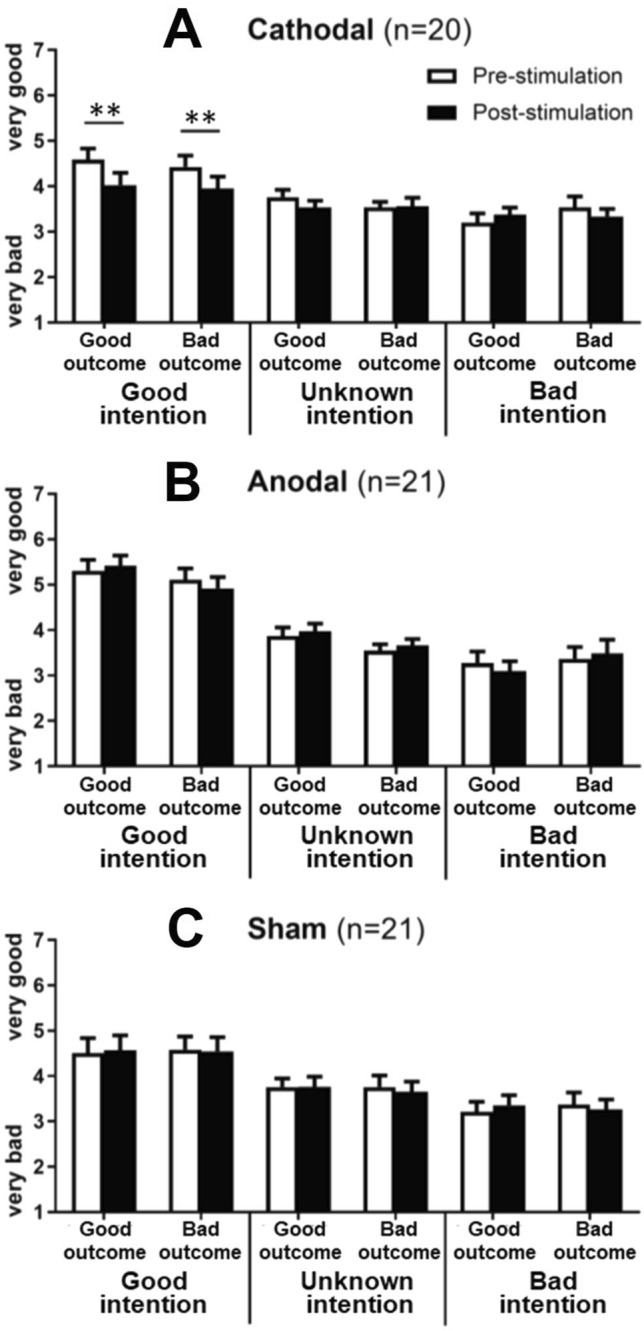
tDCS effects across experiments. Cathodal tDCS over the rTPJ diminished outcome evaluation when the intention is good. *p < 0.05, **p < 0.01.

To make sure that three groups are comparable across conditions, we first checked the demographic information. Overall, no significant differences in terms of the age were observed among the three stimulation groups, *p* = 0.805 (see Table 1). Then, to check the intention-based social judgements towards good and bad intentions/outcomes in the baseline condition, a two-way repeated measure ANOVAs with intention and outcome as within-subject factors were performed before the tDCS stimulation for each group individually. Our results revealed a significant main effect of intention attribution for each group (Cathodal: F (2, 38) = 26.66, *p* < 0.001, *η*_*p*_^2^ = 0.58, see Fig. 3A; Anodal: F (2, 40) = 39.12, *p* < 0.001, *η*_*p*_^2^ = 0.66, see Fig. 3B; Sham: F (2, 40) = 53.010, *p* < 0.001, *η*_*p*_^2^ = 0.47, see Fig. 3C) before tDCS-stimulation. Specifically, the ratings under the good intention showed the highest, followed by the unknown intention, and then the bad intention, with either of the two differ from each other. No main effect of outcome and interaction effect between intention and outcome were observed significantly, all *p* values > 0.05. These results have consistently suggested an intention-oriented attribution during social judgments across three groups regardless of the outcomes.

**Table 1:** Ages and emotion scores for tDCS groups (mean ± SD).

Measurements	Anodal	Cathodal	Sham	*F* value	*p*
Age	20.29 ± 1.35	20.40 ± 2.14	20.66 ± 2.18	0.217	0.805
State anxiety	41.38 ± 5.55	38.90 ± 5.96	38.52 ± 5.52	1.562	0.218
Positive affect	21.76 ± 7.39	21.05 ± 7.03	21.19 ± 6.36	0.061	0.941
Negative affect	18.14 ± 7.79	17.80 ± 6.86	16.81 ± 7.72	0.180	0.836
Uncomfortableness	3.10 ± 1.79	3.30 ± 2.03	2.81 ± 1.97	0.336	0.716

Then, a four-way repeated measure ANOVA was performed with intention (good vs. unknown vs. bad), outcome (good vs. bad), and phase (pre-tDCS vs. post-tDCS) as within-subject variables, stimulation group (anodal, cathodal, sham) as a between-subject variable, and the rating scores as a dependent variable. Our results have revealed a significant interaction effect among the four factors (F (4, 118) = 2.543, *p* = 0.043, *η*_*p*_^2^ = 0.079). A further two-way repeated measure ANOVA with intention and outcome as within-subject factors were performed after the tDCS stimulation for each group individually. Interestingly, the previously observed main effect of intention was disappeared after cathodal stimulation over the rTPJ compared to pre-stimulation (Cathodal: F (2, 38) = 3.80, *p* = 0.08, *η*_*p*_^2^ = 0.15, see Fig. 3A), suggesting a disruption of intention-based attribution after transient deactivation of the rTPJ. However, the main effect of intention was maintained for the anodal (Anodal: F (2, 40) = 30.95, *p* < 0.001, *η*_*p*_^2^ = 0.60, see Fig. 3B) and sham (Sham: F (2, 40) = 12.77, *p* < 0.001, *η*_*p*_^2^ = 0.39, see Fig. 3C) groups, respectively, suggesting that anodal or sham stimulation over the rTPJ didn’t impact the intention-based attribution, pointing to differential effects of anodal and cathodal tDCS on intention attribution during social judgements.

Further comparison between pre- and post-stimulation for the cathodal group were performed across three intention levels to address how cathodal stimulation modulated intention attribution. Specially, we found that the cathodal stimulation over the rTPJ largely diminished the goodness rating of both good outcome (pre vs. post: M = 4.59 ± 1.09 vs. M = 4.03 ± 1.22, *p* = 0.003, Cohen’s *d* = 0.48) and bad outcome (pre vs. post: M = 4.43 ± 1.12 vs. M = 3.95 ± 1.16, *p* = 0.007, *d* = 0.42) when the intention is good, but leaving the outcome evaluations under either the unknown (good outcome: *p* = 0.69, *d* = 0.08; bad outcome: *p* = 0.58, *d* = 0.12) and the bad intention (good outcome: *p* = 0.28, *d* = 0.25; bad outcome: *p* = 0.75, *d* = 0.07) unchanged before and after stimulation. Altogether, our results demonstrated a casual role of the rTPJ in modulating the intention-based outcome evaluation, with deactivation in the rTPJ impaired intention attribution mainly by diminishing the goodness ratings towards good/bad outcomes when the intentions are good.

Lastly, statistics on the subjective questionnaires revealed no difference on the subjective ratings towards anxiety, positive/negative affect, and uncomfortableness levels during stimulation (see Table 1, all *p* values >  0.218), suggesting that the observed modulatory effects of cathodal tDCS on the intention attribution during social judgments cannot be attributed to the changes in emotion, personal states, or the technical manipulation difference across three sessions that are induced by stimulation etc.

## Discussion

In our research, we orthogonalized good/bad intention and good/bad outcome. We found that cathodal tDCS over the rTPJ diminished the goodness ratings towards good/bad outcomes when the intentions are good and showed no difference with ratings under bad/unknown intentions. Our results provide the first evidence that deactivation in the rTPJ disrupted intention-oriented attribution mainly by lowering the goodness ratings toward outcomes when the intentions are good, but not when the intentions are bad/unknown. Our findings argue for a causal role of the rTPJ in modulating intention attribution that may rely on the nuanced intention-outcome combinations.

### Dissociation of intention and outcome

In our study, no main effect of outcome valence was observed. The judgment difference we have observed in a baseline condition are mainly driven by the intention-oriented attribution, suggesting that goodness ratings were mainly driven by intention. It is possible that participants may show strong demanding prosocial characters, thus leading to higher weighting of intention than outcome. The outcomes (good/bad) here indeed didn’t impact their self-interest as a third-party. Meanwhile, the outcome itself didn’t convey moral values or utilities^15,18,21,23^. Our work is generally in line with previous results showing intention-dominated evaluation patterns^25^.

### Comparison with previous studies

First and foremost, we have observed a largely reduced role of the intention on social judgments after inhibiting the rTPJ. Similar impairment on intention attribution was also observed in others’ studies^15,20^. However, the disruption of intention attribution in our study was mainly driven by the diminished goodness ratings when the initial intentions are good regardless of the goodness of outcomes. No modulatory effects of the tDCS on the negative intentions and negative outcomes were observed in our study. This finding is inconsistent with previous results showing tDCS effects on attempted or intentional harm, in which deactivating the rTPJ enhanced the permission of neutral or negative outcomes when the intentions were negative. Such discrepancy may come from the differences in addressing the seriousness of consequences across contexts: in previous studies, the outcomes were usually framed as socially undesirable events, such as death or serious disease that have strong and serious negative impacts^15,18,20^, while the outcomes in the present study were framed with fair or unfair monetary allocations that have on influence on self-benefits and don’t convey severe influence. Previous studies have shown stronger emotional arousal toward the same events when they are framed as negative compared to positive^37^. One recent study has specifically indicated the role of the TPJ and the TPJ-mPFC connection in modulating decision preference toward the same offers but were framed differently in a social decision task^38^. It is very likely that the impaired intention-oriented attribution shown in our study relies on the desirability of contexts. Moreover, the difference in expectation or top-down attention may also matter across contexts. In the traditional tasks, strong expectation violation may occur when the protagonist’s belief is incongruent with foreshadowing (negative intentions), but not when the proposer’s proposal is unfair. Indeed, fair proposal (positive intentions) could be even more unexpected in our current design, thus leading to stronger modulatory effects.

The second difference from previous studies is that no modulatory effects on the intention attribution were observed after activating the rTPJ via anodal tDCS. The absence of anodal tDCS effects on judgments of either good or bad intention may be due to the ceiling effect^39^. In the present study, anodal tDCS may have little benefit on the intention attribution as the rTPJ could have been sufficiently activated under socially desirable contexts. Another reason could be the individual baseline difference in rating^40^. The baseline ratings of goodness may be high enough so that the room for enhancement after anodal tDCS is small. There is one study has shown that anodal tDCS over the right posterior parietal cortex (PPC) only improved short-term memory for participants with poor baseline performance^41^.

### Context-based intention modulation

Besides the essential role for intention and outcome processing, the rTPJ was also implicated in the deployment of attention (“attention hypothesis”) to an unexpected event as firstly suggested by Buccino et al^42^. In his study, non-intended actions showed stronger activation in the rTPJ compared to intended actions but not the reverse (“intention hypothesis”)^42^, and then followed by others^43–45^. Based on our findings, though rTPJ plays a similarly disruptive role in modulating social judgments under scenarios with good intentions compared to bad intentions, such effects cannot be merely attributed to attentional mechanism or expectancy violation. The reason is an equally modulatory effect of the cathodal tDCS on the bad outcome was found as the good outcome under the good intention though the former one is more unexpected. When we bring previous studies and our current findings together, it is obvious that the cathodal tDCS over rTPJ consistently disrupted the intention-based social judgments not only by enhancing the permission of failed harms^15,20^, but also by decreasing the positive evaluations of the good/bad outcomes when the intentions are good. Such results may argue for a causal role of the rTPJ in modulating intentional attribution that is also closely relying on the contexts.

### Limitations and future directions

Our study has some limitations. Firstly, it is still unclear whether the effects we observed in the current experiment can be extended to situations in which consequences are not merely monetary and much more serious. Intentions in our study are limited to the financial fairness dimension. Our experimental design does not speak to more evil motives, such as to harm, to sabotage, and to kill. Secondly, it is possible that other brain regions apart from the rTPJ may also be modulated indirectly by tDCS. Future studies may directly measure the neural activity at the whole brain level after tDCS. Lastly, our findings need to be treated with caution and wait for further replications. Future studies may further investigate the dose effect of tDCS and the effects of other tDCS protocol parameters.

## Conclusions

We demonstrated that cathodal tDCS over the rTPJ disrupted intention-oriented attribution in social judgments mainly by reducing the goodness rating towards both good/bad outcomes when the intentions are good. Our findings argue for a causal role of the rTPJ in modulating intention-based social judgments and call for further investigation of the nuanced functions of rTPJ in social judgment.

## Data Availability

The datasets generated during and/or analyzed during the current study are available from the corresponding authors on reasonable request.
